# Successful management of recurrence of lung surgery-induced pyoderma gangrenosum after pacemaker implantation: a case report

**DOI:** 10.1186/s44215-022-00029-8

**Published:** 2023-03-30

**Authors:** Jun Muto, Ryunosuke Hase, Sho Narita, Shouhei Otsuka, Akihiro Sasaki, Tatsuya Kato

**Affiliations:** 1 Department of Thoracic Surgery, Steel Memorial Muroran Hospital, 1-45 Chiribetsu-Cho, Hokkaido, 050-0076 Japan; 2 Department of Thoracic Surgery, Hokkaido University Hospital, N14W5, Kita-Ku, Sapporo, Hokkaido, 060-8648 Japan

**Keywords:** Pyoderma gangrenosum, Lung surgery, Pacemaker implantation

## Abstract

**Background:**

Pyoderma gangrenosum begins as a painful erythema with induration, vesicles, or hemorrhagic pustules, which develops into serous ulcers. In addition to ulcerative colitis and myelodysplastic syndrome, surgical intervention may also induce this disease.

**Case presentation:**

A 90-year-old man had previously undergone video-assisted thoracic surgery left upper lobectomy for left lung cancer. Blood tests on the 6th postoperative day showed elevated levels of white blood cells and C-reactive protein. The wound appeared red and drained pus; however, the wound culture was negative for bacteria. A skin biopsy was performed on the 13th postoperative day, and the patient was diagnosed with pyoderma gangrenosum. Tacrolimus hydrate ointment was administered, symptoms gradually improved around the 18th postoperative day, and the erythematous area shrank. The patient was discharged on the 50th postoperative day.

Six months after lung surgery, a pacemaker was implanted in the left subclavian region. On the 6th postoperative day, the wound appeared reddish-brown and exudate was observed. On the 10th postoperative day, wound dehiscence was observed, and the pacemaker was removed. The patient was diagnosed with recurrent pyoderma gangrenosum and was re-treated with ointment. On the 29th postoperative day, a leadless pacemaker, which can be implanted with a small incision, was selected for treating arrhythmia. The patient was discharged 7 days after the second implantation.

**Conclusion:**

We report a recurrent pyoderma gangrenosum case following lung cancer surgery after pacemaker implantation as the second surgery, in which disease recurrence could be prevented by changing to a leadless pacemaker. Surgery and other invasive procedures should be avoided in pyoderma gangrenosum patients.

## Introduction

Pyoderma gangrenosum begins as a painful erythema with induration, vesicles, or hemorrhagic pustules, which develops into serous ulcers. In addition to ulcerative colitis and myelodysplastic syndrome, surgical intervention may also induce pyoderma gangrenosum, as in this case. In the present report, we describe successful recovery from recurrent pyoderma gangrenosum that developed after pacemaker implantation as the second surgery, in a patient who had recovered from postoperative pyoderma gangrenosum following lung cancer surgery.

## Case

The patient was a 90-year-old man diagnosed with left upper lobe lung cancer. The patient had a history of dyslipidemia, carotid artery stenosis, and hypertension. He had no history of pyoderma gangrenosum or surgery. Preoperative blood samples were obtained for TP, ALB, liver function, HbA1c, and renal function, which were normal. His oral medication included teprenone, famotidine, mecobalamin, pravastatin sodium, sennoside, brotizolam, aspirin, and cilostazol.

**Fig. 1:**
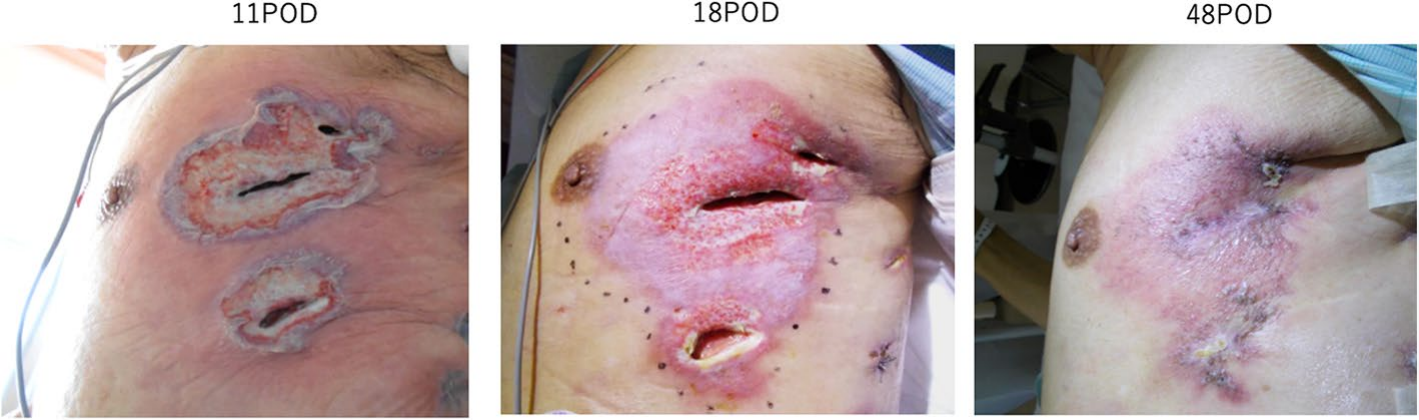
Appearance of the skin after left upper lobectomy. 11POD: Ulceration was consistent with the surgical wound. Extensive erythema was observed around the wound. Yellow necrotic tissue was observed at the abyss of the wound. 18POD: After treatment with tacrolimus ointment. Reddish shallow ulcers and erosions spread to 1.5-cm margins of the largest ulcers. The erythematous area was shrinking. 48POD: All ulcer areas are epithelialized. The open wound is closed. *POD* postoperative day

**Fig. 2:**
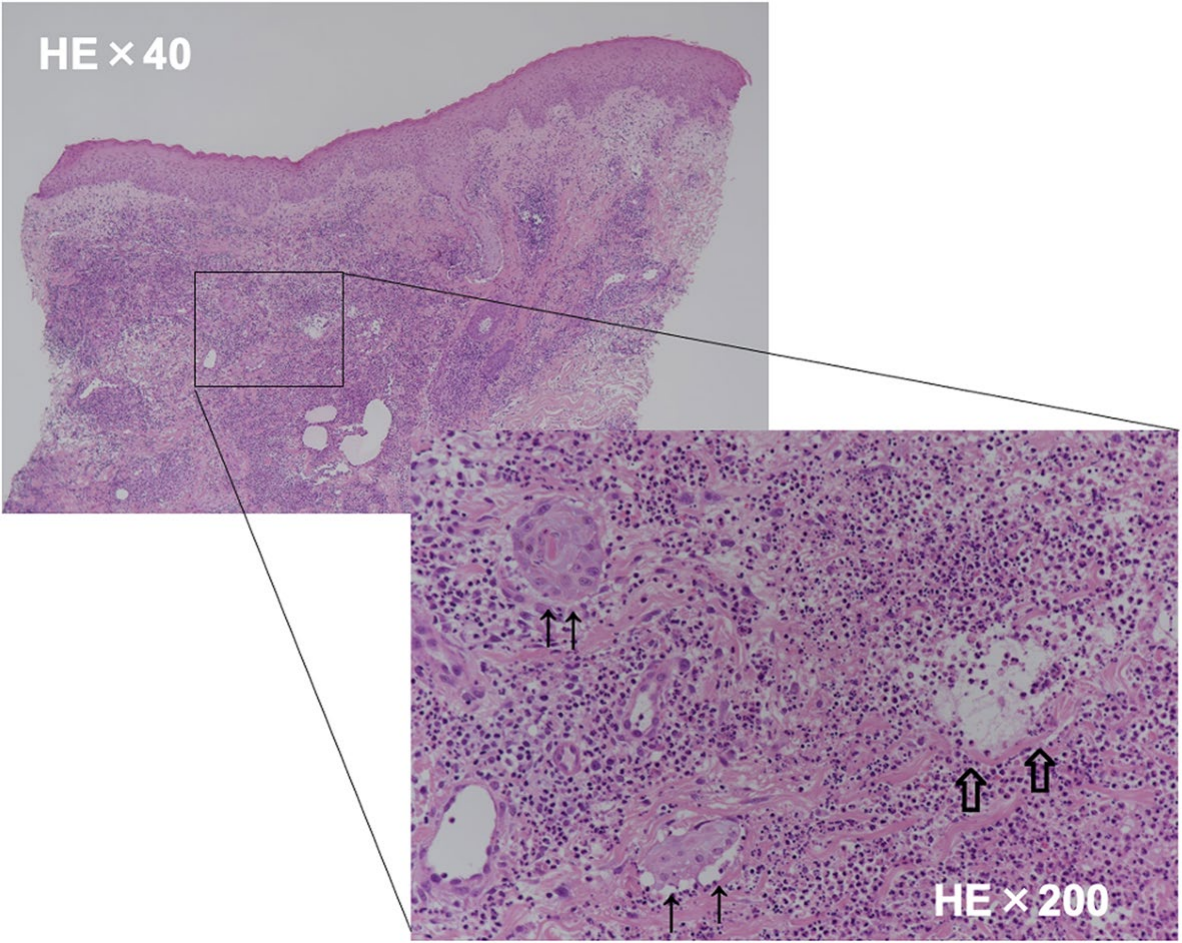
Pathological findings. Histopathological findings of the skin. The epidermis displays minor changes, but the dermis shows neutrophilic inflammation (↑) and abscess (⇧), including the follicular area. No fungi or pathogens were detected in the specimen. The patient was diagnosed with pyoderma gangrenosum

The patient had previously undergone video-assisted thoracic surgery left upper lobectomy for lung cancer. The surgery time was 223 min, and blood loss was 50 ml. The thoracic drainage tube was removed on the 3rd postoperative day (3POD). Blood tests on 6POD showed elevated levels of white blood cells and C-reactive protein. The wound was red and began to drain pus; however, the wound culture was negative for bacteria. Despite daily antibiotic treatment and wound cleaning, the wound condition worsened (Fig. 1, *left*). A skin biopsy was performed on 13POD. Pathological findings revealed necrotic collagen fibers and diffuse neutrophilic inflammatory cell infiltration between the collagen fibers. Abscess formation and stromal cells also exhibited edematous degeneration, and no fungi or pathogens were found in the specimen (Fig. 2). Based on pathological and clinical findings, the patient was diagnosed with pyoderma gangrenosum and was treated with tacrolimus hydrate ointment. His symptoms gradually improved by approximately 18POD (Fig. 1, *middle*), and the erythematous area shrank. All ulcerated areas epithelialized 7 weeks postoperatively, and the wound closed (Fig. 1, *right*). The patient was discharged on 50POD. Given the patient’s age, he was followed up without postoperative adjuvant chemotherapy. The lesions extended to the skin and subcutaneous tissue, and the muscular layer was closed and did not become pyothorax. Moreover, no gangrenous pyoderma occurred at the puncture site of the infusion. After discharge, the patient was not treated with tacrolimus.

**Fig. 3:**
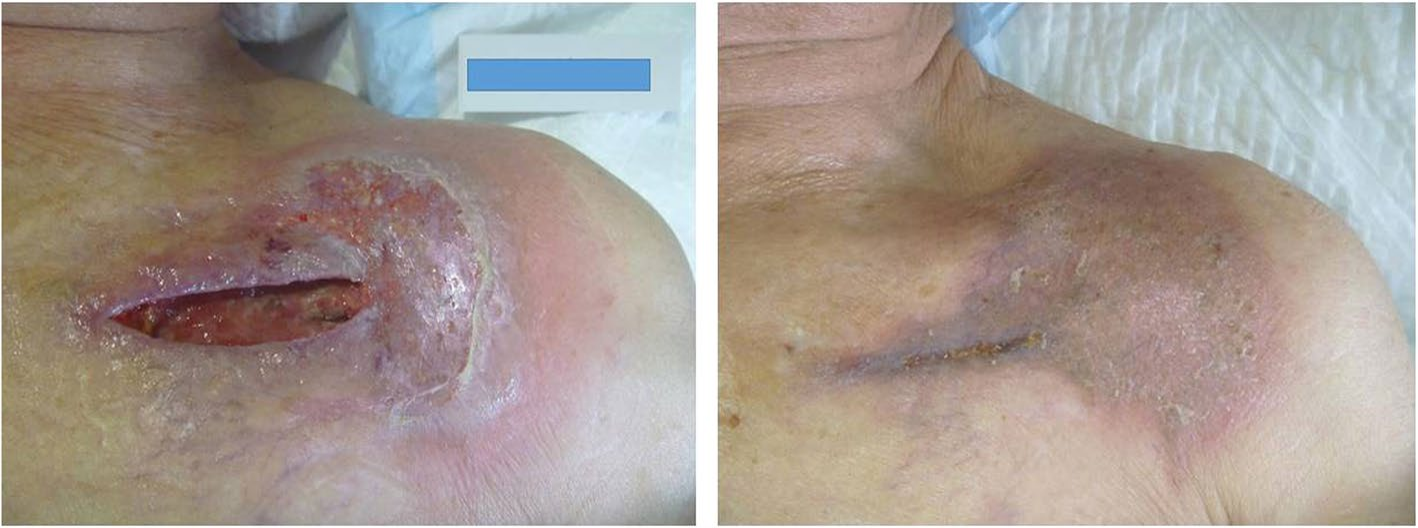
Appearance of the skin after pacemaker implantation. Left: After removal of the port, tacrolimus ointment was applied to the wound. Right: Wound after epithelialization

Six months after the lung surgery, the patient visited the cardiology department with a chief complaint of shortness of breath. Electrocardiography revealed a complete atrioventricular block, and the patient was immediately admitted for pacemaker implantation. A pacemaker was implanted in the left subclavian region. On 6POD, the wound appeared reddish-brown with exudate. On 10POD, wound dehiscence was observed, and the pacemaker was removed. The patient was diagnosed with recurrent pyoderma gangrenosum and was re-treated with ointment. On 29POD, a leadless pacemaker, which can be implanted with a small incision, was selected as the treatment option for arrhythmia. A leadless pacemaker was implanted through the right femoral vein in the groin to the right ventricle. The patient was discharged 7 days after the second implantation (Fig. 3). The patient is currently in the outpatient ward with no recurrence of lung cancer 14 months after surgery.

## Discussion

Pyoderma gangrenosum was first described by Brunsting in 1930 as a painful progressive skin ulceration of unknown cause in patients with ulcerative colitis [1]. It begins as a painful erythema with induration, vesicles, or hemorrhagic pustules, developing into serous ulcers, and is associated with various underlying systemic diseases, such as inflammatory bowel disease and aortitis [2]. Moreover, invasive surgery, as in this case, contributes to the onset of this disease [2]. The incidence of pyoderma gangrenosum is reportedly 3–10 per million per year [3]. Similar infectious diseases include gangrenous fasciitis, herpes ulcers, dermatomycosis, inflammation due to specific inflammation (i.e., tuberculosis, syphilis), and pyogenic pustulosis (i.e., caused by *Pseudomonas aeruginosa*). Similar non-infectious diseases include drug-induced tumors, systemic vasculitis, artificial injury, antiphospholipid antibody syndrome, and necrotic spider bites [4].

Postoperative pyoderma gangrenosum, which has an average onset time of 7 days after surgical intervention [5], is easily mistaken for surgical site infection at the onset, and more than 60% of cases are treated with surgical anti-inflammatory methods (i.e., wound cleaning and antibiotics) [6]. The most frequent sites of pyoderma gangrenosum are the breast and abdomen. It is relatively rare for pyoderma gangrenosum to occur after lung cancer surgery or pacemaker implantation, as in the reported case [5, 7]. In addition, pyoderma gangrenosum can sometimes occur due to stress to a site different to the one where it initially develops [8].

The diagnosis of postoperative pyoderma gangrenosum is based on the exclusion of similar diseases [9]. Bacteriological examination and skin lesion biopsy are necessary for excluding similar diseases. Pathological findings of pyoderma gangrenosum include vasculitis with aggregation of lymphocytes in biopsies of the erythematous area, immune complex deposits in the endothelium or perivascular area, and necrosis and neutrophil infiltration in the peri-ulcer area. There are no characteristic findings, but findings need to be differentiated from those that indicate wound infection [1]. In the present case, the bacterial culture of the wound was negative and the wound worsened; therefore, we suspected pyoderma gangrenosum at a relatively early stage and performed a skin biopsy.

The first choice of standard treatment for pyoderma gangrenosum is the cutaneous application of steroids or tacrolimus ointment; systemic administration of steroids or immunosuppressive agents is necessary if the effect of topical treatment is insufficient [9]. Topical tacrolimus is effective in patients with peristomal disease [10]. In this case, based on the advice of a dermatologist, tacrolimus was used as the first treatment choice, symptoms improved in response, and steroids were not used. Had tacrolimus not been effective, we intended to use steroids.

The cardiologist was *not* aware of the pyoderma gangrenosum at that time. Regarding the treatment of arrhythmia, an implantable pacemaker was the first choice because it was ideal. The size of the leadless pacemaker wound was approximately 1 cm long. In this case, the wound could be reduced in size, and the progression of pyoderma gangrenosum could be prevented by changing the pacemaker implantation to a leadless pacemaker.

We were able to prevent the development of pyoderma gangrenosum at the puncture site in this patient by re-implanting a leadless pacemaker, which could be implanted with a small wound. In that sense, we believe that this was “successful management.”

## Conclusion

Herein, we report a case of recurrent pyoderma gangrenosum that occurred after lung cancer surgery following pacemaker implantation as the second surgery, in which recurrence could be managed by changing to a leadless pacemaker. Surgery and other invasive procedures should be avoided in patients with pyoderma gangrenosum.

## Data Availability

Data sharing is not applicable to this article, as no datasets were generated or analyzed during the current study.

## References

[1] Powell FC, Schroeter AL, Su WP, Perry HO. Pyoderma gangrenosum: a review of 86 patients. Q J Med. 1985;55:173–86. 3889978

[2] Coady K. The diagnosis and treatment of pyoderma gangraenosum. J Wound Care. 2000;9:282–5. 11933343 10.12968/jowc.2000.9.6.25991

[3] Ruocco E, Sangiuliano S, Gravina AG, Miranda A, Nicoletti G. Pyoderma gangrenosum: an updated review. J Eur Acad Dermatology Venereol. 2009;23:1008–17. 10.1111/j.1468-3083.2009.03199.x 19470075

[4] Callen JP, Jackson JM. Pyoderma gangrenosum: an update. Rheum Dis Clin North Am. 2007;33:787–802. 18037117 10.1016/j.rdc.2007.07.016

[5] Tolkachjov SN, Fahy AS, Cerci FB, Wetter DA, Cha SS, Camilleri MJ. Postoperative pyoderma gangrenosum: a clinical review of published cases. Mayo Clin Proc. 2016;91:1267–79. 27489052 10.1016/j.mayocp.2016.05.001

[6] Tolkachjov SN, Fahy AS, Wetter DA, Brough KR, Bridges AG, Davis MDP, et al. Postoperative pyoderma gangrenosum (PG): the Mayo Clinic experience of 20 years from 1994 through 2014. J Am Acad Dermatol. 2015;73:615–22. 26209218 10.1016/j.jaad.2015.06.054

[7] Frey P, Akret C, Irles D, Dompnier A, Bing AC. Pyoderma gangrenosum complicating a permanent pacemaker implantation: a case report and literature review. Eur Heart J Case Rep. 2020;4:1–7. 32352047 10.1093/ehjcr/ytaa049 PMC7180522

[8] Yamamoto H, Sugimoto S, Otani S, Toyooka S. Postoperative pyoderma gangrenosum exacerbated by granulocyte-colony stimulating factor after lung cancer surgery. Jpn J Clin Oncol. 2017;47:991–2. 28981742 10.1093/jjco/hyx121

[9] Quist SR, Kraas L. Treatment options for pyoderma gangrenosum. J Dtsch Dermatol Ges. 2017;15:34–40. 28140549 10.1111/ddg.13173

[10] Lyon CC, Stapleton M, Smith AJ, Mendelsohn S, Beck MH, Griffiths CE. Topical tacrolimus in the management of peristomal pyoderma gangrenosum. J Dermatolog Treat. 2001;12:13–7. 12171681 10.1080/095466301750163518

